# Genomic Regions 10q22.2, 17q21.31, and 2p23.1 Can Contribute to a Lower Lung Function in African Descent Populations

**DOI:** 10.3390/genes11091047

**Published:** 2020-09-04

**Authors:** Héllen Fonseca, Thiago M. da Silva, Mariana Saraiva, Meddly L. Santolalla, Hanaisa P. Sant’Anna, Nathalia M. Araujo, Natália P. Lima, Raimon Rios, Eduardo Tarazona-Santos, Bernardo L Horta, Alvaro Cruz, Mauricio L. Barreto, Camila A. Figueiredo

**Affiliations:** 1 Programa de Pós Graduação em Imunologia (PPGIm), Instituto de Ciências da Saúde, Universidade Federal da Bahia (UFBA), Salvador 40140-100, BA, Brazil; hellenffreitas@gmail.com (H.F.); mari.md07@gmail.com (M.S.); raimonrios@gmail.com (R.R.); 2 Departamento de Ciências Biológicas, Universidade Estadual do Sudoeste da Bahia, Jequié 45206-190, BA, Brazil; thiago@uesb.edu.br; 3 Departamento de Genética, Ecologia e Evolução, Instituto de Ciências Biológicas, Universidade Federal de Minas Gerais, Belo Horizonte 31270-901, Brazil; meddly.santolalla@gmail.com (M.L.S.); hanaisaa@hotmail.com (H.P.S.); nathalia.matta.araujo@gmail.com (N.M.A.); edutars@gmail.com (E.T.-S.); 4 Programa de Pós-Graduação em Epidemiologia, Universidade Federal de Pelotas, Pelotas 96020-220, Rio Grande do Sul, Brazil; natyplima@hotmail.com (N.P.L.); blhorta@gmail.com (B.L.H.); 5 ProAR, Faculdade de Medicina, Universidade Federal da Bahia (UFBA), Salvador 40060-330, BA, Brazil; cruz.proar@gmail.com; 6 Centro de Integração de dados e Conhecimentos para Saúde (CIDACS), Fiocruz, Salvador 41745-715, BA, Brazil; mauricio@ufba.br; 7 Departamento de Bio-Regulação, Instituto de Ciências da Saúde, Universidade Federal da Bahia (UFBA), Salvador 40110-902, BA, Brazil

**Keywords:** lung function decline, admixture mapping strategy, African ancestry

## Abstract

Accumulated evidence supports the contribution of genetic factors in modulating airway function, especially ancestry. We investigated whether genetic polymorphisms can affect lung function in a mixed Brazilian child population using the admixture mapping strategy through RFMix software version 1.5.4 (Stanford University, Stanford, CA, USA), followed by fine mapping, to identify regions whereby local African or European ancestry is associated with lung function measured by the forced expiratory volume in the first second (FEV_1_)/forced vital capacity (FVC) ratio, an indicator of airway obstruction. The research cohort included 958 individuals aged 4 to 11 years enrolled in the SCAALA (Social Change, Asthma, Allergy in Latin America) Program. We identified that African ancestry at 17q21.31, 10q22.2, and 2p23.1 regions was associated with lower lung function measured by FEV_1_/FVC *p* < 1.9 × 10^−4^. In contrast, European ancestry at 17q21.31 showed an opposite effect. Fine mapping pointed out 5 single nucleotide polymorphisms (SNPs) also associated in our replication cohort (rs10999948, rs373831475, rs8068257, rs6744555, and rs1520322). Our results suggest that genomic regions associated with ancestry may contribute to differences in lung function measurements in African American children in Brazil replicated in a cohort of Brazilian adults. The analysis strategy used in this work is especially important for phenotypes, such as lung function, which has considerable disparities in terms of measurements across different populations.

## 1. Introduction

The diagnosis of asthma and other conditions affecting the respiratory tract is based mainly on the observation of clinical symptoms along with pulmonary function test evaluation. In this sense, spirometry is an essential tool for the diagnosis and classification of the severity of such diseases [1]. The forced expiratory volume in the first second (FEV_1_)/forced vital capacity (FVC) ratio is used as a criterion for airflow obstruction. A diagnosis of airflow obstruction supports the diagnosis of asthma [2].

The pulmonary function of children and adolescents is influenced by different factors, such as height, age, sex, weight, ethnicity, and intrinsic factors associated with the development process [3]. The main risk factors for impaired pulmonary function are prematurity, early-life respiratory tract infections [4], and environmental factors, such as tobacco smoke exposure [5]. However, some evidence supports that genetic factors are also important in determining susceptibility to airway obstruction [6]. According to the genome-wide association study (GWAS) Catalog, more than 21,000 associations were reported for lung function considering the most diverse populations worldwide [7].

Global differences in pulmonary function measurements and between distinct ethnic groups have been noted independent of asthma symptoms and Chronic obstructive pulmonary disease (COPD) [8]. It has been reported that varying degrees of African, Native American, and European ancestry present in the African American population could influence complex traits [9]. Inverse relations have been found between African ancestry and FEV_1_ and FVC measures [10], even after adjustment for anthropometrics and socioenvironmental variables [11]. Moreover, the higher the African ancestry contribution among asthmatics, the greater the severity of asthma as defined by lower pre-FEV_1_ values [10].

Almost all of these studies, however, were conducted on North American populations, and little is known about the relationship between African ancestry and pulmonary function in the Latin American context. Brazilians are one of the most heterogeneous populations in the world, with a great contribution of African ancestors to their admixture process [12]. Furthermore, if the observed relationship between African ancestry and spirometric measures is due to genetic factors underlying African ancestry, the admixture mapping strategy could help to elucidate the genetic variants implicated in the ethnic-racial inequalities extensively reported for lung function. This method uses an admixed population to map ancestry-associated genomic regions related to complex phenotypes by testing the correlation between the phenotype and the ancestry of contiguous small chromosome segments along the genome. Such an approach, by reducing the number of statistical tests to be performed, improves power to detect an association when compared to GWAS [13], and may be complemented by second-phase fine-mapping strategies if high density data are available.

Taken together, the present study aims to identify genetic variants that may contribute to pulmonary function differences in an admixed Brazilian population by admixture mapping strategy, which is especially suitable for this approach.

## 2. Materials and Methods

### 2.1. Study Design and Populations Studied

This study was based on data from EPIGEN-Brazil, one of the largest Latin American initiatives at the interface of human genomics, public health, and computational biology [14]. Two cohorts were included in this analysis, the Social Change, Asthma, Allergy in Latin America (SCAALA) Salvador cohort (discovery cohort) [15] and the “1982 Pelotas birth cohort” (replication cohort) [16].

The discovery population included 958 unrelated children between 4 and 11 years old from SCAALA (Social Change, Asthma, Allergy in Latin America) living in the city of Salvador (State of Bahia, Brazil) with a population of approximately 2.9 million habitants (IBGE, 2019) [17].

The children were recruited in early childhood to participate in a prospective study that aimed to measure the impact of a sanitation program in the city of Salvador on child morbidity [15].

The “1982 Pelotas birth cohort” study was conducted in Pelotas, a city in South Brazil, with 214,000 urban inhabitants. Throughout 1982, the three maternity hospitals in the city were visited daily and births were recorded, corresponding to 99.2% of all births in the city. Pulmonary function measurements were performed at the 2012–2013 follow-up, at which time participants were approximately 30 years old [16].

Potential confounding variables, such as sex, age, Body mass index (BMI), and tobacco exposure characteristics, were collected for the two cohorts (Table S1). Individuals with missing data for the main variates used in the linear regression models were excluded. For both cohorts, related individuals were excluded from the analysis. In brief, we estimated kinship coefficients for each possible pair of individuals from each cohort, using the method implemented in the REAP software v 1.2 (University of Washington, Seattle, WA, USA) (Related Estimation in Admixed Populations) [18]. We considered a pair of individuals as related if the estimated kinship coefficient between them was ≥0.1. This cutoff includes second-degree relatives, such as a person’s uncle/aunt, nephew/niece, grandparent/grandchild, or half- sibling and any closer pair of relatives.

The EPIGEN protocol was approved by Brazil’s National Research Ethics Committee (CONEP, protocol # 15895, Brasília, Brazil). Kehdy et al. (2015) estimated the proportions of African, European, and Native American ancestries for each individual of each cohort [12] using the ADMIXTURE software v 1.3.0 (University of California, Oakland, CA, USA) [19], and we used those estimates in the present study (Table S1).

### 2.2. Spirometric Measurements

Spirometry was performed in accordance with the recommendations of the American Thoracic Society [20], as described by SMA Matos et al. (2011) [21]. The percent predicted values of forced vital capacity (FVC), forced expiratory volume in 1 s (FEV_1_), and FEV_1_/FVC ratio were measured and then calculated in both discovery and replication cohorts in accordance with the Brazilian standard curve [22]. The bronchodilation test was performed by inhaling 200 mg salbutamol, with spirometry being performed 15 min after administration of the bronchodilator. 

### 2.3. Genomic DNA Extraction and Genotyping

Genotyping was performed using standardized commercial panel 2.5 HumanOmni Beadchip, including some ancestry informative markers (AIMs), and is currently available from Illumina (www.illumina.com), as detailed in Kehdy et al. (2015) [12]. For quality control (QC), we excluded single nucleotide polymorphisms (SNPs) with minor allele frequency (MAF) < 0.005, and we defined a minor allele count (MAC) > 20 for such analyses. Markers with genotyping call rates of less than 0.98 and individuals with missing data for more than 10% of SNPs were excluded.

### 2.4. Local Ancestry Inferences

For local ancestry inferences, we used RFMix software v 1.5.4 (Stanford University, Stanford, CA, USA) [23]. Additionally, we used parental population data from 1000 Genome Project populations CEU and IBS (Europeans); Africans from Ghana, Botswana and Gambia (from NIH, Sarah Tishkoff lab and 1000 Genome Project, Gouveia et al. 2020); and eight Native American populations (Quechuas, Ashaninkas, Shimaas, Aymaras from Tarazona-Santos group Laboratory of Human Genetic Diversity (LDGH) dataset and Matsiguengas, Queros, Uros, and Moches from the Peruvian National Health Institute–INS, Lima, Peru) [24]. RFMix uses a conditional random field parameterized by random forests trained on reference panels, learning from the admixed samples to autocorrect phasing errors and improve local ancestry inferences [23]. To run RFMix, we fixed the number of generations since the admixture event (parameter-G) to 20 (~500 years) and the number of trees to generate per random forest (parameter-*t*) to 500. Inferences were performed in window lengths (parameter -w) of 0.2 cM. All other parameters present in RFMix were set as default. For RFMix, we considered only the windows for which ancestry was inferred with a posterior probability >0.90.

### 2.5. Relationship between Lung Function and Individual Ancestry

To assess the effect of individual admixture (European, African, and Native American) on lung function, we used a linear regression analysis, including age, sex, and BMI as covariates. All statistical analyses were performed using the R platform v 3.6.0 (University of Auckland, Auckland, New Zealand) [25] and PLINK software v 1.9 (Boston, MA, USA) [26].

### 2.6. Admixture Mapping

We performed admixture mapping in the SCAALA cohort using RFMix inferences as stated above. RFMix uses the genetic location of SNPs to divide the chromosome into W contiguous disjoint windows. We tested the association of Lung function (FEV_1_/FVC ratio percentage) separately for each local ancestry (African, European, and Native American) across the genome using linear regression models. The three models were adjusted by age, sex, BMI, and global African ancestry. We used an additive model that considers the number of inferred African, European or Native American ancestry copies (0, 1 or 2) carried by an individual for each window [27]. We used PLINK software [26] to perform linear regressions. A total of 16,237 windows were included.

To establish a significance threshold for admixture mapping, accounting for multiple testing, we followed the method of Shriner et al. (2011), based on the estimation of the effective number of tests (ENT) for each chromosome for each individual [28]. To do this, the method fits an autoregressive model to the vector of local ancestries (0, 1, or 2 chromosomes of given ancestry) and evaluates the spectral density at frequency zero with the package coda for R. Then, we used the estimated ENT to obtain a Bonferroni p-value threshold for significance as 0.05 divided by ENT. Genome-wide *p*-value thresholds were obtained for each ancestry in each dataset (Table 1). We defined a significant *p*-value < 1.7 × 10^−4^ (for African ancestry); *p*-value < 1.89 × 10^−4^ (for European ancestry) and *p*-value < 4.85 × 10^−5^ (for Native American ancestry).

### 2.7. Imputation, Fine Mapping, Annotation

Only the significant admixture mapping peaks were followed-up for fine-mapping analysis. We used an EPIGEN-Brazil dataset imputed with IMPUTE2 [29], focusing on ±1 Mb centered in the most significant window of each admixture mapping hit. We imputed our dataset with a reference panel that merged the public reference panel data from the 1000 Genome Project and 270 individuals from EPIGEN (90 of each cohort) genotyped for 4.3 million SNPs as a reference panel, and we considered only SNPs imputed with an info score quality metric >0.8 [14].

The genotyped and imputed SNP genotypes for all markers were tested for association with lung function using the same linear regression models used for admixture mapping in the SCAALA cohort. We excluded SNPs with minor allele frequency <0.005 and (MAC) ≤20 for these analyses. After QC, the regions 17q21.31 (22,945 variants), 2p23.1 (12,042 variants), 10q22.2 (6681 variants), and 4p15.2 (1007 variants) remained in the SCAALA cohort. In the Pelotas cohort, 17q21.31 (24,131 variants), 2p23.1 (12,766 variants) and 10q22.2 (15,654 variants) remained. We considered significant associations with *p*-values lower than those obtained for the admixture mapping peaks. Fine mapping results were plotted using the LocusZoom tool [30]. We used Ensembl to annotate associated genetic variants [31] and the Haploview v 4.1 software (Cambridge, MA, USA) was used to calculate the degree of confidence in the *r*^2^ value [32]. Finally, the SNPs with significant *p*-values found in the SCAALA cohort were tested in the replication cohort using phenotypes equal to the discovery cohort and following the same methodology (linear regression analysis) and QC.

## 3. Results

### 3.1. Study Population

The online Supplementary Table S1 summarizes the sociodemographic characteristics, lung function measurements and other variables of the studied populations (SCAALA and 1982 Pelotas birth cohort) [15,16]. The outcomes evaluated were the percentages obtained to FEV_1_, FVC and FEV_1_/FVC ratio for each patient. The Global Initiative for Asthma (GINA) and The Global Initiative for COPD (GOLD) use the FEV_1_/FVC ratio as a parameter to assess the presence of airflow limitation. The latest international guidelines for lung function assessment have recommended that the ideal way to identify airway obstruction is to compare the observed measurement to the lower limit of normality (LLM) [33].

The mean values for FEV_1_ and FVC before and after bronchodilator administration can be seen for the SCAALA and Pelotas cohorts. The average African, European and Native American ancestries were0.512 ± 0.13, 0.425 ± 0.13, and 0.063 ± 0.029, respectively, for the SCAALA cohort. On the other hand, the Pelotas cohort had a strong influence of European ancestry and a low influence of African and Native American descent represented by the respective ancestry averages (0.77 ± 0.20, 0.155 ± 0.192, and 0.07 ± 0.04).

### 3.2. Admixture Mapping and Fine Mapping for Lung Function

**Figure 1:**
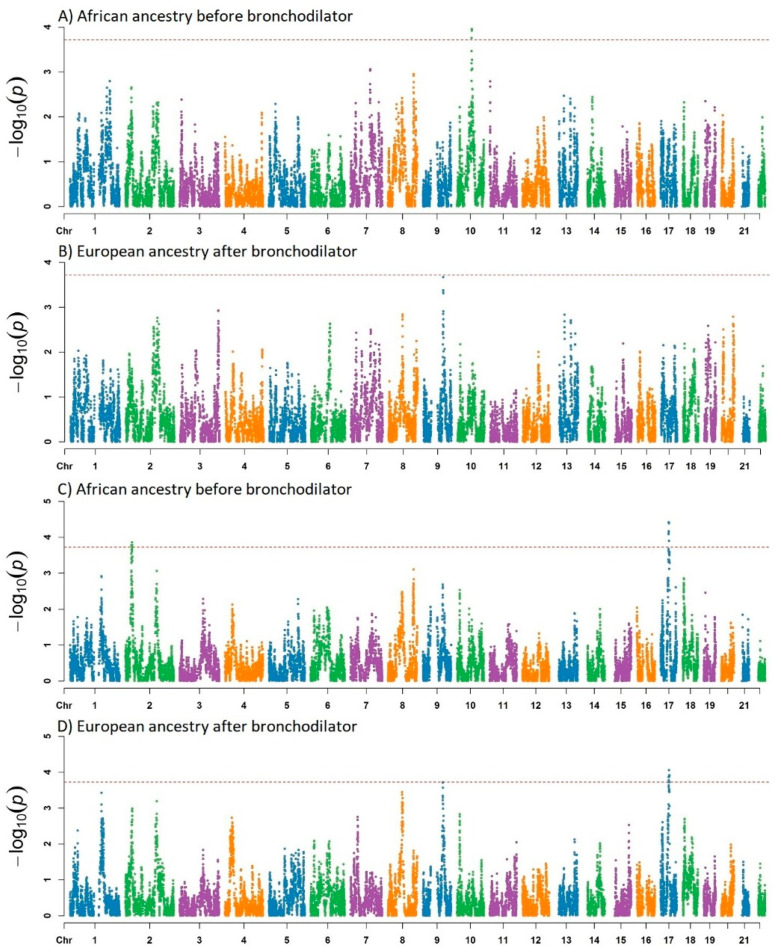
Manhattan plot of the admixture mapping (AM) for lung function for African and European ancestry in the SCAALA population (*n* = 958 children). (Figure 1): Association testing for lung function FEV_1_/FVC ratio percentage and African ancestry (**A**,**C**) before and after the bronchodilator, respectively. African ancestry AM shows negative significant peaks in 10q22.1 (**A**) and negative significant peaks in 17q21.31 and 2p23.1 (**C**). Testing for lung function FEV_1_/FVC and European ancestry (**B**,**D**) before and after the bronchodilator, respectively. European ancestry AM analysis shows a positive associated peak at 17q21.31 (**D**). Analysis of 17q21.31 and 2p23.1 regions were also associated with lower lung function (Table 1, Figure 1C). In turn, European ancestry at 17q21.31 showed the opposite, that is, higher FEV_1_/FVC ratio values (Table 1, Figure 1D). Native American ancestry at the 4p15.2 region was also associated with higher values for FEV_1_/FVC ratio (Table 1 and Figure S1).

We performed admixture mapping analysis for the three continental ancestries in Brazilians (African, European, and Native American). For the SCAALA cohort, four significant admixture mapping peaks were found for the percent predicted values of FEV_1_/FVC in 10q22.2, 17q21.31 2p23.1, and 4p15.2, considering as threshold the *p*-values previously indicated in Methods (Table 1 and Figure S1). Figure 1 shows the Manhattan plots for the admixture mapping results based on RFMix chromosome ancestry inferences. African ancestry at 10q22.2 regions was associated with lower levels of lung function as measured by the percent of FEV_1_/FVC before bronchodilator (Table 1, Figure 1A). The Manhattan plots for Native American ancestry are shown in Figure S1. Quantile-quantile (QQ) plots for the percent predicted values of FEV_1_/FVC before and after bronchodilator administration are shown in Figure S2.

After imputing the associated chromosomal regions by admixture mapping, fine mapping was performed to find SNPs significantly associated with the %FEV_1_/FVC ratio in the SCAALA cohort (Table S2). Importantly, in fine mapping for the 4p15.2 locus related to Native American ancestry; no SNP was significantly associated (considering a *p*-value < 4.85 × 10^−5^).

Admixture mapping was also performed for the percent FEV_1_ and FVC values measured before and after bronchodilator use. We found three significant admixture mapping peaks in 3q29, 7q31.1, and 15q22.2 (Table S3). African ancestry in 3q29, 7q31.1 and 15q22.2 were associated with lower levels of FEV_1_ and FVC, while European ancestry in 15q22.2 was associated with higher measures of FVC. 

**Figure 2:**
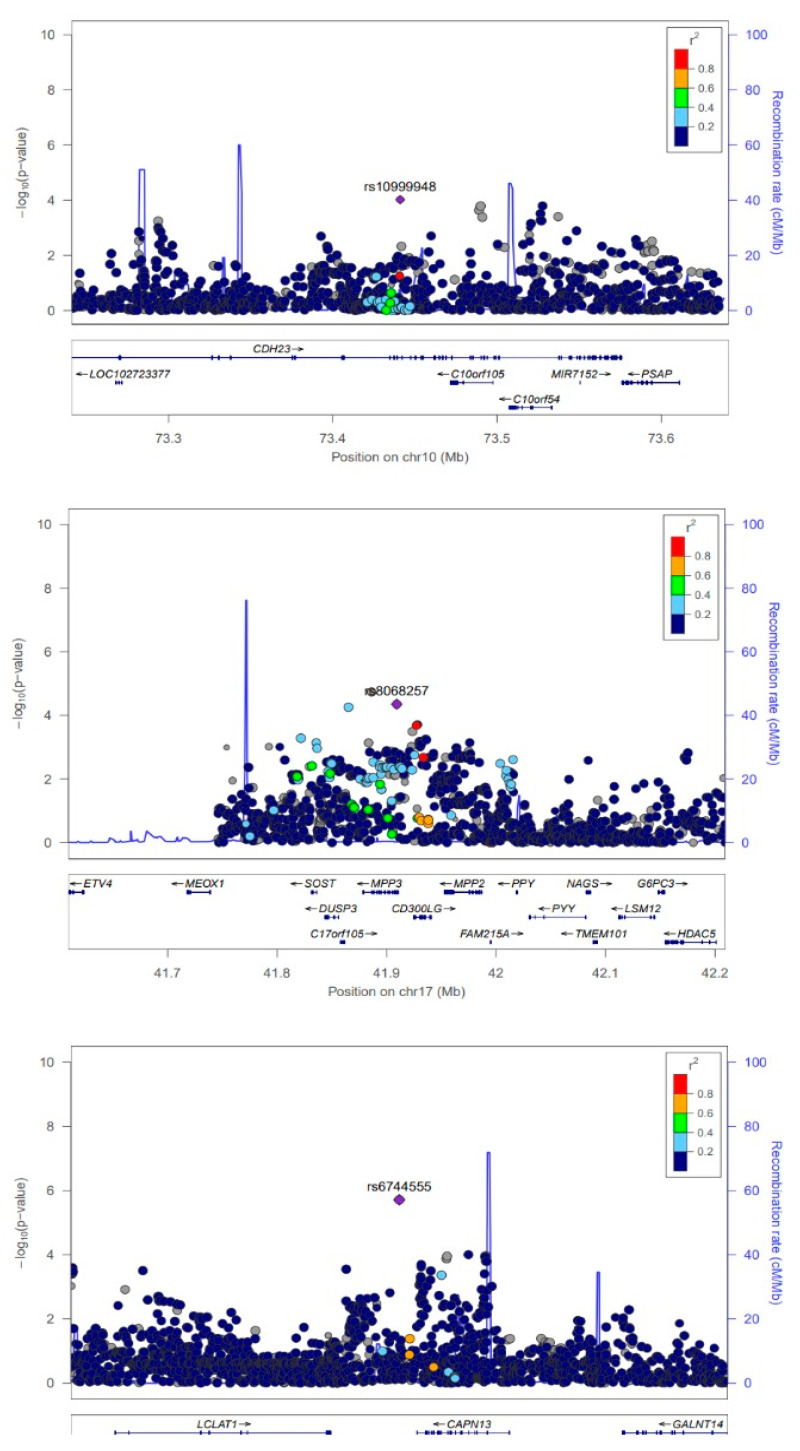
LocusZoom plot of the region around 10q22.2 (rs10999948), 17q21.31 (rs8068257), and 2p23.1 (rs6744555) among children from the SCAALA Cohort in Salvador, Brazil. The region includes genotyped and imputed variants from 1000 Genomes phase III. Pairwise r^2^ values are from hg19/1000 Genomes European data (November 2014 release).

The LocusZoom plot shows the regions including genotyped and imputed variants from 1000 Genomes phase 3 next to the most associated variants for the SCAALA cohort in Figure 2, using Pairwise r^2^ values from hg19/1000 Genomes European data (November 2014 release) and the r^2^ values from hg19/1000 Genomes African data is shown in Figure S3. The Figure S4 shows the LocusZoom plot of the same regions for the Pelotas cohort (hg19/1000 Genomes European data).

Fine mapping for FEV_1_ and FVC was also performed for the chromosomal regions indicated in the admixture analysis (Table S4). An association was found only for FVC measurements, where markers in the 3q29 and 7q22.3 regions were associated with lower lung function.

Analysis were also performed considering the differences between the post- and pre-bronchodilator values for the FEV_1_/FVC ratio (Table S5).

### 3.3. Replication Analysis 

We attempted to replicate the findings related to %FEV_1_/ FVC in 2846 individuals from a Pelotas birth cohort study. Five SNPs previously associated in Fine mapping to the SCAALA cohort were associated in the Pelotas cohort tested by linear regression considering a significant *p*-value < 0.05. (Table 2). The associations between rs10999948 and rs6744555 and FEV_1_/FVC identified in the Pelotas cohort were in the same direction in the SCAALA cohort (β= −0.725 and β= −0.675). In addition, rs373831475, rs8068257, and rs1520322 presented the opposite direction of the results obtained in the SCAALA cohort (β = 0.674, β = 0.67, and β = 0.412, respectively).

We considered adjusting the analyses adding asthma to both cohorts. The variable asthma influenced the association of SNPs rs373831475, rs8068257, rs6744555, and rs1520322 in the Pelotas cohort, with a lack of statistical significance (*p*-value > 0.05).

## 4. Discussion

Here, we used genomic data from a cohort of children in northeastern Brazil to identify loci associated with pulmonary function through the admixture mapping approach followed by a fine mapping strategy. We found three significant admixture mapping peaks (10q22.2, 17q21.31, and 2p23.1) where African local ancestry was associated mainly with lower pulmonary function.

Several studies have reported that variants in region 17q21 are associated with an increased risk for asthma and infant wheeze in different worldwide populations [34]. The 2p23 region also associated with asthma severity [35]. Moreover, Wain et al. 2015, demonstrated an association between 17q21.31 and lower FEV_1_ in smokers and non-smokers [36]. Despite the close relationship between asthma and lower lung function, few studies have evaluated the association of those genomic regions with spirometric measurements in American populations. None of the SNPs pointed out in our fine mapping were previously associated with lung function, and the fact that most studies taking place in European populations may have contributed to this.

Different studies report that African ethnicity is correlated with lower lung function (measured as FEV_1_ and FVC) in several populations, which has also been reported in Brazil, but no difference has been observed in the FEV_1_/FVC ratio [37]. A study comparing spirometric reference values for Caucasians and African Americans showed that African American individuals had FEV_1_ and FVC below the lower limit of normal [38]. Harik-Khan et al. 2001 have also demonstrated that the racial difference in lung function is partially explained by a chromosome segment in African Americans and low socioeconomic indicators explain a small proportion of this racial difference [39]. Lower FEV_1_ and FVC measures, in the absence of disease, suggest reduced lung size or growth. A lower FEV_1_/FVC ratio, however, indicates airflow limitation. Therefore, a lower FEV_1_/FVC ratio associated with genetic determinants may be a relevant risk factor for these conditions.

None of the SNPs found here were previously associated with pulmonary function or respiratory tract diseases. rs10999948 A>G are in the Cadherin-23 (CDH23) gene. It was associated with lower lung function attributed to FEV_1_/ FVC in the two studied cohorts (SCAALA β = −1.572; Pelotas β = −0.725). Cadherin-23 (CDH23) forms a large family of proteins often involved in calcium-dependent cellular adhesion. It is involved in the conversion of a mechanical stimulus to an electrical signal and is crucial to our ability to hear and maintain balance [40]. Different missense mutations in CDH23 have been reported to cause autosomal recessive nonsyndromic hearing loss (ARNSHL) or progressive hearing loss. Moreover, polymorphisms in the CDH1 gene (included in the family of E-cadherins) have been described, being associated with airway remodeling, inflammation, and forced expiratory volume in 1 s (FEV_1_) decline in asthma patients [41]. However, the mechanism whereby CDH23 may contribute to lower pulmonary function is not fully understood.

In the SCAALA cohort, although African ancestry in the 17q21.31 region was associated with a risk for lower pulmonary function, European ancestry in the same region was associated with higher pulmonary function. It is important to note that the SCAALA and Pelotas cohorts (replication cohort) present very different ancestral genetic contributions. While the SCAALA cohort had a strong influence of African ancestry, and presented an average of African and European ancestry corresponding to 0.512 ± 0.13 and 0.425 ± 0.13, respectively, the Pelotas cohort had a strong influence of European ancestry and a low influence of African descent represented by the respective ancestry averages (0.77 ± 0.20 and 0.155 ± 0.192). The 17q21.31 region pointed to variants (rs8068257 A>G and rs373831475), in the membrane palmitoylated protein (MPP3) gene were also associated in our analysis in opposite directions for the SCAALA and Pelotas cohorts (as seen in Table 2).

Most likely, the changes in the direction of the association for these SNPs in Pelotas are due to differences in the linkage disequilibrium (LD) pattern compared to that observed in the SCAALA cohort. Indeed, the LD analysis around the SNPs that were oppositely associated with lung function in the SCAALA and Pelotas cohorts reveals important differences in relation to the extent of the LD for these regions between the two populations (Figure S5). This suggests that the phasing of the LD (i.e., in coupling or repulsion) between these SNPs and the true causal variants associated with lung function may also occur differently in the two populations, which would explain the changes observed in the effect of these SNPs between both.

According to the NCBI, MPP3 is a member of a family of membrane-associated proteins termed MAGuKs (membrane-associated guanylate kinase homologs). There are reports that MAGuKs regulate neutrophil polarity, with functional implications for both inflammatory diseases and infections [42]. Mutations of MAGuKs are linked to many human diseases, including cancers, psychiatric disorders, and intellectual disabilities. In addition, there are reports that MPP3 and DAL-1 (differentially expressed in adenocarcinoma of the lung protein) interact with TSLC1 (tumor suppressor in lung cancer-1) and that these genes may play an important role in a TSG (Tumor-suppressor Genes) cascade that regulates cell growth. The loss of function of the TSLC1 cascade may promote metastasis. There is a significant correlation between loss of expression and methylation of TSLC1 and DAL-1 in lung cancer cell lines [43]. No previous study has shown an association of polymorphisms in this gene with pulmonary function or diseases affecting the respiratory tract. However, these divergences in the direction of the associations between SCAALA and Pelotas cohort for the (rs8068257 A>G) suggest that the differences in degrees of ancestry in the two study populations may influence the risk for bronchial obstruction measured through FEV_1_/ FVC.

We also obtained a hit at the calpain 13 gene (CAPN13) for rs1520322 G>A. This variant was also associated in opposite directions between the SCAALA (β = −1.23) and Pelotas (β = 0.412) cohorts. Calpains are a class of intracellular, calcium-dependent cysteine (Cys) proteases [44]. Although the physiological function is still unclear, there are reports of mutations in the Calpains gene associated with altered apoptosis and disturbance of the IkB/NF-kB pathway. Genetic variation or production deficiency of calpain were also associated with type 2 diabetes mellitus, production of eosinophil chemoattractants, and eosinophil accumulation and activation [45]. In addition, it is important to emphasize that not all members of this family of proteins have their functions properly identified, as is the case for CAPN13. More detailed studies are needed to investigate the impact of variants on this gene in the population studied herein, given the high frequency of the top SNPs on the CAPN13 gene.

There are no reports associated with the variants (rs373831475 and rs6744555 C>A) also associated in our study; thus, functional studies must be conducted to explore this possible and previously unrecognized pathophysiological pathway. The genes mentioned here interact with several other genes that participate in important pathways for cell function and inflammatory processes, demonstrated in Figure S6 obtained by pathwaycommons.org.

There are several limitations to this study. The SCAALA cohort consists of a population-based study of children with high African ancestry, and the phenotypes evaluated here are not readily accessible in any other cohort with genomic data available or with a similar genetic structure to our discovery set. Brazil is a very large country, and, despite the Native American, African, and European ancestry contributing to the formation of this population, there are significant differences in the contribution of each of these ancestries in different territories, as observed in the two populations addressed in this study, one with a high African contribution (SCAALA) and the other with high European contribution (Pelotas birth cohort) [12]. Moreover, these are individuals of different ages (children versus young adults), which is reflected in the investigated outcome. Nevertheless, given these particularities of the studied cohorts, through replication analysis, we were able to confirm the role of the three chromosomal regions 10q22.2, 17q21.31, and 2p23.1 in lung function.

Other studies in this area would also be improved by greater comparability between cases and controls for pulmonary function, preferably among subjects with pediatric asthma. Understanding the mechanisms whereby genetics modulates pulmonary function is very important to improve new intervention strategies, including prevention of diseases in more vulnerable individuals and early treatment. This study also highlights how non-European and mixed populations are a source of a new genetic variant associated with the genetic architecture of lung function in a population of Brazilian children.

## Figures and Tables

**Table 1:** Peak regions indicated by admixture mapping for lung function measured by the %forced expiratory volume in the first second (FEV_1_)/forced vital capacity (FVC) ratio, an indicator of airflow limitation, before and after bronchodilator administration, among children from the Social Change, Asthma, Allergy in Latin America (SCAALA) Cohort in Salvador, Brazil.

Trait	Chr Regions	Chr Position	Initial Window Marker	Final Window Marker	Ancestry	Effect (β)	*p*-Value
FEV_1_/FVC (before bronchodilator)	10q22.1–10q22.2	10:74052899	rs16929751	rs73272395	African	−1.27	1.09 × 10^−04^
		10:73717457	rs11498014	rs111781439	African	−1.26	1.18 × 10^−04^
		10:73527047	rs1867982	rs115465907	African	−1.27	1.73 × 10^−04^
FEV_1_/FVC (after bronchodilator)	17q21.31	17:41085242	rs116644941	rs12951528	African	−1.17	3.82 × 10^−05^
		17:40114544	rs4796750	rs7502710	African	−1.17	3.82 × 10^−05^
		17:41689336	rs74961000	rs1107748	African	−1.16	4.04 × 10^−05^
		17:40261545	rs12600570	rs77641795	African	−1.14	6.72 × 10^−05^
		17:40919959	rs35381342	rs76847100	African	−1.12	7.46 × 10^−05^
		17:39902271	rs7219088	rs4594300	African	−1.12	8.13 × 10^−05^
	17q21.31	17:41689336	rs74961000	rs1107748	European	1.12	8.70 × 10^−05^
		17:43368600	rs16939953	rs1724385	European	1.09	1.21 × 10^−04^
		17:42872361	rs730818	rs713101	European	1.09	1.32 × 10^−04^
		17:41774588	rs115305838	rs1684668	European	1.09	1.33 × 10^−04^
		17:40114544	rs4796750	rs7502710	European	1.10	1.34 × 10^−04^
FEV_1_/FVC (after bronchodilator)	2p23.1	2:33733554	rs6761582	rs4287749	African	−1.089	1.40 × 10^−04^
		2:34018767	rs62150613	rs75291994	African	−1.083	1.64 × 10^−04^
		2:30145791	rs12714294	rs77336532	African	−1.072	1.65 × 10^−04^
	4p15.2	4:21669818	rs150467258	rs4621420	Nat. A.	2.688	4.86 × 10^−06^
		4:21488871	rs358580	rs4697227	Nat. A.	2.493	9.93 × 10^−06^
		4:21339971	rs16870863	rs114908241	Nat. A.	2.476	1.18 × 10^−05^
		4:22133556	rs115891762	rs9992463	Nat. A.	2.436	1.77 × 10^−05^
		4:20637946	rs1465522	rs1156304	Nat. A.	2.465	2.01 × 10^−05^
		4:21701301	rs2167246	rs115214367	Nat. A.	2.466	2.035 × 10^−05^
		4:21245276	rs77753664	rs71607066	Nat. A.	2.355	3.08 × 10^−05^
		4:20535717	rs3775816	rs936360	Nat. A.	2.407	3.90 × 10^−05^

Admixture mapping in 10q22.2, 17q21.31, 2p23.1, and 4p15.2 for lung function (ratio FEV_1_/FVC) before and after bronchodilator use for three ancestralities (African, European, and Native American (Nat. A.)) in the SCAALA population (*n* = 958 children). Significant *p*-value < 1.89 × 10^−04^ (European ancestry), *p*-value < 1.7 × 10^−04^ (African ancestry) and *p*-value < 4.85 × 10^−5^ (for Native American ancestry). Position according to the National Center for Biotechnology Information (NCBI), GRCh37.p13. https://www.ncbi.nlm.nih.gov/snp/. Analysis adjusted by age, sex, Body mass index (BMI) category, and global African ancestry covariates. FEV_1_, forced expiratory volume in 1 s; FVC, forced vital capacity; effect (β), regression coefficient; Chr, Chromosome. The initial/final window marker corresponds to the interval between the initial and final single nucleotide polymorphism (SNPs) of the associated chromosomal windows defined by RFMix. Nat. A., Native American.

**Table 2:** Fine mapping significant associations for %FEV_1_/FVC ratio identified through imputed variants from 1000 Genomes phase 3 next. Analysis obtained by linear regression showing only the associated common SNPs for the SCAALA and 1982 Pelotas birth cohorts.

Cohort	Trait	CHR	SNP	BP	A1	A2	MAF	β	CI (Min)	CI (Max)	*p*-Value	β (2)	*p*-Value (2)	β (3)	*p*-Value (3)	Gene
SCAALA	FEV_1_/FVC (before bronchodilator)	10	rs10999948	73440988	G	A	0.223	−1.572	−2357	−0.786	9.51 × 10^−05^	−1.609	6.639 × 10^−05^	−1.61	6.62 × 10^−05^	CDH23
	FEV_1_/FVC (after bronchodilator)	17	rs373831475	41886161	A	ATCTTC	0.221	−1.388	−2.02	−0.756	1.83 × 10^−05^	−1.38	2.165 × 10^−05^	−1.37	2.43 × 10^−05^	MPP3
		17	rs8068257	41909216	G	A	0.268	−1.252	−1.85	−0.654	4.44 × 10^−05^	−1.252	4.69 × 10^−05^	−1.24	5.43 × 10^−05^	MPP3
		2	rs6744555	30929681	A	C	0.091	−2.083	−2.93	−1.23	1.94 × 10^−06^	−2.094	1.872 × 10^−06^	−2.09	1.95 × 10^−06^	NONE
		2	rs1520322	31008331	A	G	0.433	−1.23	−1.76	−0.697	6.78 × 10^−06^	−1.227	7.707 × 10^−06^	−1.22	7.83 × 10^−06^	CAPN13
PELOTAS	FEV_1_/FVC (before bronchodilator)	10	rs10999948	73440988	G	A	0.192	−0.725	−1.23	−0.215	5.31 × 10^−03^	−0.597	4.86 × 10^−03^			CDH23
	FEV_1_/FVC (after bronchodilator)	17	rs373831475	41886161	A	ATCTTC	0.071	0.674	−0.001	1.35	2.78 × 10^−02^	0.537	3.58 × 10^−02^			MPP3
		17	rs8068257	41909216	G	A	0.086	0.67	0.028	1.312	4.08 × 10^−02^	0.558	1.84 × 10^−02^			MPP3
		2	rs6744555	30929681	A	C	0.083	−0.675	−1.276	−0.074	2.70 × 10^−02^	−0.487	0.051			NONE
		2	rs1520322	31008331	A	G	0.273	0.411	0.024	0.798	3.72 × 10^−02^	0.346	2.67 × 10^−02^			CAPN13

Abbreviations: FEV_1_, forced expiratory volume in 1 s; FVC, forced vital capacity; Chr, chromosome; SNP, single nucleotide polymorphism; A1, minor allele (effect allele); A2, major allele; MAF, minor allele frequency corresponding to SCAALA/Pelotas cohort; CI, confidence interval; β (2)/*p*-value (2), adjusted by age, sex, BMI category, global African ancestry and asthma covariates; β (3)/*p*-value (3), adjusted by age squared, sex, BMI category, global African ancestry, and asthma covariates in SCAALA cohort. For Pelotas replication, statistical significance was evaluated at *p*-value < 0.05 at the SNP level only.

## Supplementary Materials

The following are available online at https://www.mdpi.com/2073-4425/11/9/1047/s1, Table S1: Characteristics of the population according to pulmonary function and variables included in this study; Table S2: Fine mapping significant associations for FEV_1_/FVC ratio in SCAALA cohort identified through genotyped and imputed variants from 1000 Genomes phase 3 next. Analysis obtained by linear regression; Table S3: Peak regions indicated by admixture mapping in 3q29, 7q31.1, and 15q22.2 for FVC and FEV_1_ among children from the SCAALA Cohort in Salvador, Brazil; Table S4: Fine mapping significant associations identified through linear regression for (%FVC before and after bronchodilator) among children from the SCAALA Cohort in Salvador, Brazil; Table S5: Peak regions pointed by admixture mapping for differences between the values measured after and before the bronchodilator for the %FEV_1_/FVC ratio, among children from the SCAALA Cohort in Salvador, Brazil; Figure S1: Manhattan plot of the admixture mapping (AM) for lung function for American Native ancestry in the SCAALA population (*n* = 958 children). Association testing for lung function FEV_1_/FVC before bronchodilator (A) and after bronchodilator (B). American Native ancestry AM analysis shows a positive associated peak in 4p15.2 (B). Analysis using linear regression adjusted by age, sex, BMI category, and global African ancestry covariates. Figure S2: Quantile-quantile (QQ) plots for the percent predicted values of FEV_1_/FVC before and after bronchodilator use corresponding to the data shown in the Manhattan plot. (Figure 1). A and B (African ancestry); C and D (European ancestry); E and F (American Native ancestry). Figure S3. LocusZoom plot of the region around 10q22.2 (rs10999948), 17q21.31 (rs8068257), and 2p23.1 (rs6744555) among children from the SCAALA Cohort in Salvador, Brazil. The region includes genotyped and imputed variants from 1000 Genomes phase III. Pairwise r^2^ values are from hg19/1000 Genomes African data (November 2014 release). Figure S4. LocusZoom plot of the region around 17q21.31, 2p23.1, and 10q22.2 for the Pelotas Cohort. The region includes genotyped and imputed variants from 1000 Genomes phase III. For 17q21.31 (24,131 variants included, Reference SNP rs8068257), 2p23.1 (12,766 variants included, Reference SNP rs6744555) and 10q22.2 (15,654 variants included, Reference SNP rs10999948). Pairwise r^2^ values are from hg19/1000 Genomes European data (November 2014 release). Figure S5: Linkage Disequilibrium (LD) plot with r^2^ values generated using HaploView 4.1 (Broad Institute, Cambridge, MA, USA) for the top SNPs in SCAALA (A, B, and C) and Pelotas (D, E, and F) cohorts. (A),(D) 17q21.31 locus—close to the SNP rs8068257; (B),(E) 17q21.31 locus—close to the SNP rs373831475; (C)(F) locus 2p23.1—next to SNPs rs6744555 and rs1520322. Figure S6: Interaction pathways of the membrane palmitoylated protein (MPP3), Cadherin-23 (CDH23), and calpain 13 gene (CAPN13) genes obtained by pathwaycommons.org.
