# Supplementary material for: Genomic Regions 10q22.2, 17q21.31, and 2p23.1 Can Contribute to a Lower Lung Function in African Descent Populations
Source: Genes (Basel). 2020 Sep 4;11(9):1047. doi: 10.3390/genes11091047 (PMC7565985; doi:10.3390/genes11091047)
Supplement: Supplementary file 1 [file genes-11-01047-s001.zip › Table S5.pdf]

**Table S5: Peak regions pointed by Admixture mapping for differences between the values measured after and before the bronchodilator for the %$FEV_1$/FVC ratio, among children from the SCAALA Cohort in Salvador, Brazil.**

| Trait | Chr regions | Chr Position | Initial window marker | Final window marker | Ancestry | Effect (β) | *p*-value |
|---|---|---|---|---|---|---|---|
| PERCENTAGE OF THE $FEV_1$/FVC RATIO CHANGE BETWEEN THE POSTERIOR (POST) AND PREVIOUS (PRE) VALUE OF THE BRONCHODILATOR | 7p11.2 | 7:54147523 | rs7787727 | rs68106607 | European | 1.23 | 6.54e-05 |
| | | 7:54622117 | rs73118033 | rs2163636 | European | 1.22 | 8.20e-05 |
| | | 7:53230291 | rs62450933 | rs17135869 | European | 1.21 | 1.01e-04 |
| | | 7:54769296 | rs10269438 | rs10278304 | European | 1.19 | 1.25e-04 |
| | | 7:53664058 | rs11238275 | rs17646091 | European | 1.18 | 1.28e-04 |
| | | 7:55002632 | rs2330915 | rs11977660 | European | 1.17 | 1.78e-04 |
| | | 7:55248787 | rs10241326 | rs111306717 | European | 1.16 | 1.83e-04 |
| PERCENTAGE OF THE $FEV_1$/FVC RATIO CHANGE BETWEEN THE POSTERIOR (POST) AND PREVIOUS (PRE) VALUE OF THE BRONCHODILATOR | 17q21.2-q21.31 | 17:41085242 | **rs116644941** | **rs12951528** | European | 0.94 | 5.15e-05 |
| | | 17:41689336 | **rs74961000** | **rs1107748** | European | 0.94 | 5.28e-05 |
| | | 17:40261545 | **rs12600570** | **rs77641795** | European | 0.93 | 6.04e-05 |
| | | 17:40919959 | **rs35381342** | **rs76847100** | European | 0.92 | 6.67e-05 |
| | | 17:41774588 | rs115305838 | rs1684668 | European | 0.91 | 8.65e-05 |
| | | 17:42344015 | rs933167 | rs4792937 | European | 0.90 | 1.24e-04 |
| | | 17:40114544 | **rs4796750** | **rs7502710** | European | 0.89 | 1.28e-04 |
| | | 17:42055918 | rs12953033 | rs115901854 | European | 0.87 | 1.76e-04 |
| | | 17:42433865 | rs9898394 | rs76809556 | European | 0.87 | 1.80e-04 |

Admixture mapping in 7p11.2 and 17q21.2 - q21.31 for differences between the values measured after and before the bronchodilator for the percentage $FEV_1$/FVC ratio used for European ancestry in SCAALA population (n=958 children). significant *p*-value <1.89e-04. Position according to the NCBI, GRCh37.p13. https://www.ncbi.nlm.nih.gov/snp/. Analysis adjusted by age, sex, BMI category, global African ancestry and percentage $FEV_1$/FVC ratio (PRE bronchodilator) covariates. No significant results for African ancestry **Abbreviations:** $FEV_1$, forced expiratory volume in 1 s; FVC, forced vital capacity; Effect (**β**), regression coefficient. Chromosomal regions in bold were the same ones associated with the analysis in Table 1.
