# Supplementary material for: Genomic Regions 10q22.2, 17q21.31, and 2p23.1 Can Contribute to a Lower Lung Function in African Descent Populations
Source: Genes (Basel). 2020 Sep 4;11(9):1047. doi: 10.3390/genes11091047 (PMC7565985; doi:10.3390/genes11091047)
Supplement: Supplementary file 1 [file genes-11-01047-s001.zip › Table S1.pdf]

Table S1: Characteristics of the population according to pulmonary function and variables included in this study.

Characteristics	SCAALA cohort (Total n=958)	PELOTAS cohort (Total n=2846)
**Sex**	**(n=958)**	**(n=2846)**
Female	521 (54.38%)	1442 (50.7%)
Male	437 (45.62%)	1404 (49.3%)
**Age in years**		
(Mean±SD)	(9 ± 1.7)	(30 ± 0.34)
**BMI**	**(n=958)**	**(n=2840)**
Slimness	129 (13.46%)	51 (1.8%)
Normal	671 (70.04%)	1135 (39.96%)
Overweight	158 (16.5%)	983 (34.62%)
Obesity	-	671 (23.62%)
**Asthma**	**(n=958)**	**(n=2770)**
No	731 (76.3%)	2106 (76.02%)
Yes	227 (23.7%)	664 (23.98%)
**Previous Hospitalization**	**(n=958)**	
No	893 (93.21%)	-
Yes	65 (6.79%)	-
**Smokers**		**(n=2843)**
No	-	506 (17.8%)
Yes	-	676 (23.8%)
No information	-	1661 (58.4%)
**Smokers at home**	**(n=958)**	**(n=2620)**
No	707 (73.8%)	1998 (76.26%)
Yes	251 (26.2%)	622 (23.74%)
**European Ancestry**		
Min-Max	0.064 - 0.851	0.08 - 0.99
(Mean±SD)	(0.425 ± 0.13)	(0.77 ± 0.20)
**African Ancestry**		
Min-Max	0.085 - 0.928	0.00001 - 0.879
(Mean±SD)	0.512 ± 0.13	0.155 ± 0.192

**Native Ancestry**		
Min-Max	0.004 - 0.192	0.00001 - 0.353
(Mean±SD)	0.063 ± 0.029	0.07 ± 0.04
**FVC before bronchodilator (L)**		
**(Min-Max)**	(0.99 - 3.93)	(0.57 - 7.31)
(Mean±SD)	(1.86 ± 0.47)	(4.18 ± 0.917)
**FVC after bronchodilator (L)**		
**(Min-Max)**	(0.90 - 3.97)	(1.58 - 7.11)
(Mean±SD)	(1.9 ± 0.48)	(4.17 ± 0.90)
**FEV_1 before bronchodilator (L)**		
**(Min-Max)**	(0.86 - 3.22)	(0.57 - 5.98)
(Mean±SD)	(1.67 ± 0.39)	(3.43 ± 0.74)
**FEV_1 after bronchodilator (L)**		
**(Min-Max)**	(0.79 - 3.29)	(1.14 - 5.95)
(Mean±SD)	(1.74 ± 0.41)	(3.52 ± 0.74)
**FEV_1/FVC before bronchodilator (L)**		
**(Min-Max)**	(0.69 - 1.10)	(0.43 -1)
(Mean±SD)	(0.97 ± 0.07)	(0.82 ± 0.06)
**FEV_1/FVC after bronchodilator (L)**		
**(Min-Max)**	(0.62 - 1.13)	(0.49 – 1)
(Mean±SD)	(0.99 ± 0.06)	(0.84 ± 0.05)

Abbreviations: FEV_1, Forced expiratory volume in 1 s; FVC, Forced vital capacity; SD, standard deviation, L , Liters.
