# Supplementary material for: Genomic Regions 10q22.2, 17q21.31, and 2p23.1 Can Contribute to a Lower Lung Function in African Descent Populations
Source: Genes (Basel). 2020 Sep 4;11(9):1047. doi: 10.3390/genes11091047 (PMC7565985; doi:10.3390/genes11091047)
Supplement: Supplementary file 1 [file genes-11-01047-s001.zip › Table S2.pdf]

Table S2: Fine mapping significant associations for FEV_1/FVC ratio in SCAALA cohort identified through genotyped and imputed variants from 1000 Genomes phase 3 next. Analysis obtained by linear regression.

Trait	CHR	SNP	BP	A1	A2	MAF	effect (β)	CI (min)	CI (max)	*p*-value
**FEV_1/FVC (before bronchodilator)**	10	rs10999948	73440988	G	A	0.223	-1.572	-2.357	-0.7863	9.516e-05
	10	rs16929777	74102476	G	A	0.17	-1.638	-2.42	-0.8565	4.344e-05
	10	rs10400019	74109459	C	T	0.17	-1.636	-2.418	-0.8536	4.509e-05
	10	rs11000440	74662104	A	G	0.43	-1.228	-1.837	-0.6182	8.475e-05
	10	rs75970382	74672581	T	C	0.014	-4.81	-7.229	-2.391	1.04e-04
	10	rs16930172	74684417	G	A	0.42	-1.215	-1.827	-0.6023	1.08e-04
	10	rs3747865	73527676	A	G	0.107	1.897	0.9163	2.879	1.60e-04
**FEV_1/FVC (after bronchodilator)**	17	rs66507706	43819413	GGA	G	0.231	-1.259	-1.839	-0.6791	2.292e-05
	17	rs4414533	43809844	T	C	0.242	-1.269	-1.87	-0.6674	3.867e-05
	17	rs1989480	43820893	G	T	0.226	-1.221	-1.804	-0.6383	4.378e-05
	17	rs6503444	43783370	C	T	0.371	-1.051	-1.595	-0.5071	1.61e-04
	17	rs373831475	41886161	A	ATCTTC	0.221	-1.388	-2.02	-0.7565	1.837e-05
	17	rs9909488	43339759	G	C	0.384	-1.171	-1.718	-0.625	2.903e-05
	17	rs8080227	42949992	T	C	0.095	-1.698	-2.575	-0.8203	1.588e-04
	17	rs8068257	41909216	G	A	0.268	-1.252	-1.85	-0.654	4.442e-05
	2	rs6744555	30929681	A	C	0.091	-2.083	-2.935	-1.23	1.94e-06
	2	rs72800126	30923304	C	T	0.096	-1.993	-2.839	-1.146	4.546e-06
	2	rs5830186	31008900	G	GGT	0.433	-1.235	-1.766	-0.7048	5.702e-06
	2	rs1520322	31008331	A	G	0.433	-1.23	-1.763	-0.6975	6.783e-06
	2	rs72800132	30932269	G	A	0.092	-1.963	-2.816	-1.11	7.251e-06
	2	rs72800133	30932270	G	A	0.092	-1.963	-2.816	-1.11	7.251e-06
	2	rs7594957	31005623	G	C	0.436	-1.166	-1.695	-0.6369	1.721e-05
	2	rs73925204	30039677	T	C	0.147	-1.624	-2.363	-0.8853	1.81e-05
	2	rs73922299	30026431	A	G	0.142	-1.607	-2.359	-0.854	3.131e-05
	2	rs6732918	31009479	A	G	0.441	-1.127	-1.662	-0.5919	3.971e-05
	2	rs72867184	31008039	A	C	0.215	-1.359	-2.008	-0.7095	4.455e-05
	2	rs7604473	30029827	A	G	0.143	-1.527	-2.284	-0.7703	8.232e-05
	2	rs55968228	30920846	C	A	0.175	-1.374	-2.058	-0.6897	8.912e-05
	2	rs73922300	30030441	T	G	0.171	-1.406	-2.108	-0.7045	9.217e-05
	2	rs10495780	31008142	C	T	0.219	-1.29	-1.935	-0.6441	9.668e-05
	2	rs58406898	30992821	G	A	0.220	1.264	0.6303	1.897	9.9e-05
	2	rs147556359	31008765	TA	T	0.218	-1.281	-1.927	-0.6351	1.088e-04
	2	rs56228971	30029187	G	A	0.147	-1.463	-2.202	-0.7251	1.094e-04
	2	rs17010279	31007974	C	A	0.218	-1.277	-1.922	-0.6316	1.127e-04
	2	rs10495781	31008246	C	G	0.218	-1.273	-1.919	-0.6273	1.195e-04
	2	rs13017304	30534867	T	A	0.322	1.091	0.5288	1.653	1.52e-04
	2	rs77682199	30037108	C	T	0.139	-1.478	-2.241	-0.7162	1.528e-04
	2	rs1402050	31008887	G	A	0.219	-1.25	-1.897	-0.6044	1.58e-04
	2	rs56136454	31009400	G	A	0.214	-1.264	-1.918	-0.6102	1.61e-04

Analysis adjusted by age, sex, BMI category, and global African ancestry covariates. **Abbreviations:** FEV_1, Forced expiratory volume in 1 s; FVC, Forced vital capacity; Chr, chromosome; SNP, Single nucleotide polymorphism; A1, minor allele (effect allele); A2, major allele; MAF, Minor allele frequency corresponding to SCAALA cohort; effect (β), regression coefficient; CI, confidence interval.
