# Supplementary material for: Genomic Regions 10q22.2, 17q21.31, and 2p23.1 Can Contribute to a Lower Lung Function in African Descent Populations
Source: Genes (Basel). 2020 Sep 4;11(9):1047. doi: 10.3390/genes11091047 (PMC7565985; doi:10.3390/genes11091047)
Supplement: Supplementary file 1 [file genes-11-01047-s001.zip › Table S3.pdf]

Table S3: Peak regions indicated by Admixture mapping in 3q29, 7q31.1 and 15q22.2 for FVC and FEV_1 among children from the SCAALA Cohort in Salvador, Brazil

Trait	Chr regions	Chr position	Initial window marker	Final window marker	Ancestry	Effect (β)	*p*-value
FVC (before bronchodilator)	3q29	3:193103028	rs7632075	rs10446356	African	0.439	1.33e-04
		3:193144406	rs2367612	rs6805912	African	-2.236	1.49e-04
		3:192831322	rs60337897	rs60294829	African	-2.236	1.75e-04
		3:72293527	rs59992807	rs7642076	European	2.345	1.64e-04
FVC (after bronchodilator)	7q31.1	7:107471433	rs28601279	rs2528651	African	2.441	8.88e-05
		7:107634989	rs6963842	rs10226708	African	2.378	1.34e-05
		7:107307206	rs2701688	rs6466182	African	2.356	1.42e-04
		7:106647929	rs2177862	rs74760552	African	2.353	1.51e-04
FEV_1 (before bronchodilator)	15q22.2	15:59509663	rs11638386	rs28516046	African	-2.159	1.94e-04

Position according to the NCBI, GRCh37.p13. https://www.ncbi.nlm.nih.gov/snp/. **Abbreviations:** FEV_1, forced expiratory volume in 1 s; FVC, forced vital capacity; Chr. chromossome
