# Supplementary material for: Genomic Regions 10q22.2, 17q21.31, and 2p23.1 Can Contribute to a Lower Lung Function in African Descent Populations
Source: Genes (Basel). 2020 Sep 4;11(9):1047. doi: 10.3390/genes11091047 (PMC7565985; doi:10.3390/genes11091047)
Supplement: Supplementary file 1 [file genes-11-01047-s001.zip › Table S4.pdf]

Table S4: Fine mapping significant associations identified through linear regression for (%FVC before and after bronchodilator) among children from the SCAALA Cohort in Salvador, Brazil.

Trait	Chr region	SNP	A1	A2	MAF	Ancestry	β	CI (min)	(CI) (max)	*p*-value	β (2)	*p*-value (2)	Gene	Freq AFR	Freq EUR
FVC (before bronchodilator)	3q29	rs8180090	G	A	0.31	African	-2.36	-3.59	-1.14	1.66 e-04	-2.375	1.58 e-04	*ATP13A5*	A: 56% G: 44%	A: 90% G: 10%
	3q29	rs7650114	A	G	0.083	African	-3.89	-5.93	-1.86	1.84e-04	-3.871	2.04 e-04	*ATP13A4*	G: 84% A: 16%	G: 100% A: 0%
FVC (after bronchodilator)	7q22.3	rs2536503	A	G	0.42	African	-2.29	-3.48	-1.09	1.77e-04	-2.307	1.64 e-04	*PRKAR2B*	G: 30% A: 70%	G: 88% A: 12%

Analysis adjusted by age, sex, BMI category, and global African ancestry covariates. Inclusion criteria: MAF > 0.005, genotyping rate 98%. **Abbreviations**: FVC, forced vital capacity; Chr, chromosome; SNP, single nucleotide polymorphism; A1, minor allele (effect allele); A2, major allele; CI, confidence interval; Freq, frequency. **β** (2), *p*-value (2), adjusted by age squared, sex, BMI category, global African ancestry covariates.
