# Supplementary figures and images for: Genomic Regions 10q22.2, 17q21.31, and 2p23.1 Can Contribute to a Lower Lung Function in African Descent Populations
Source: Genes (Basel). 2020 Sep 4;11(9):1047. doi: 10.3390/genes11091047 (PMC7565985; doi:10.3390/genes11091047)

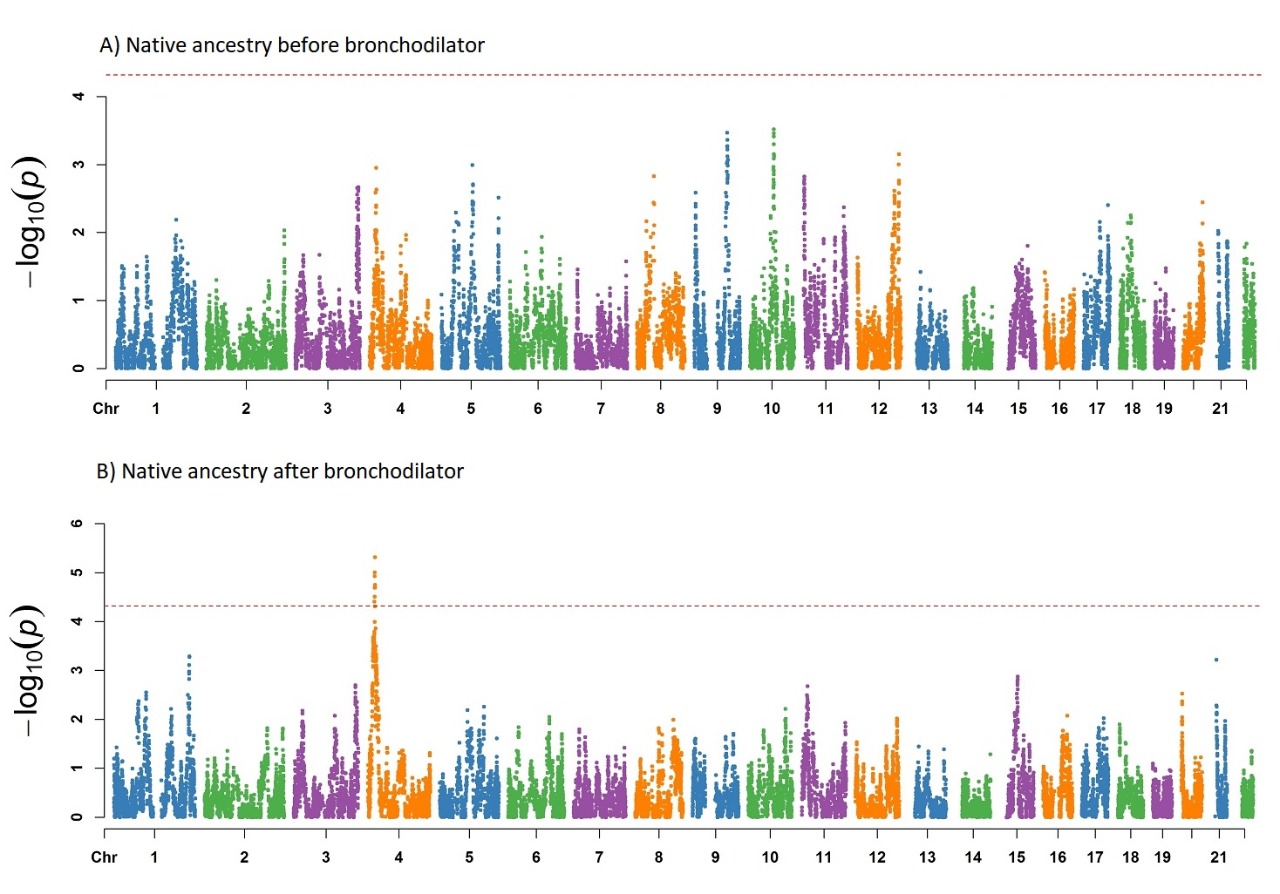

Supplement: Supplementary file 1 [file genes-11-01047-s001.zip › Figure S1.jpeg]

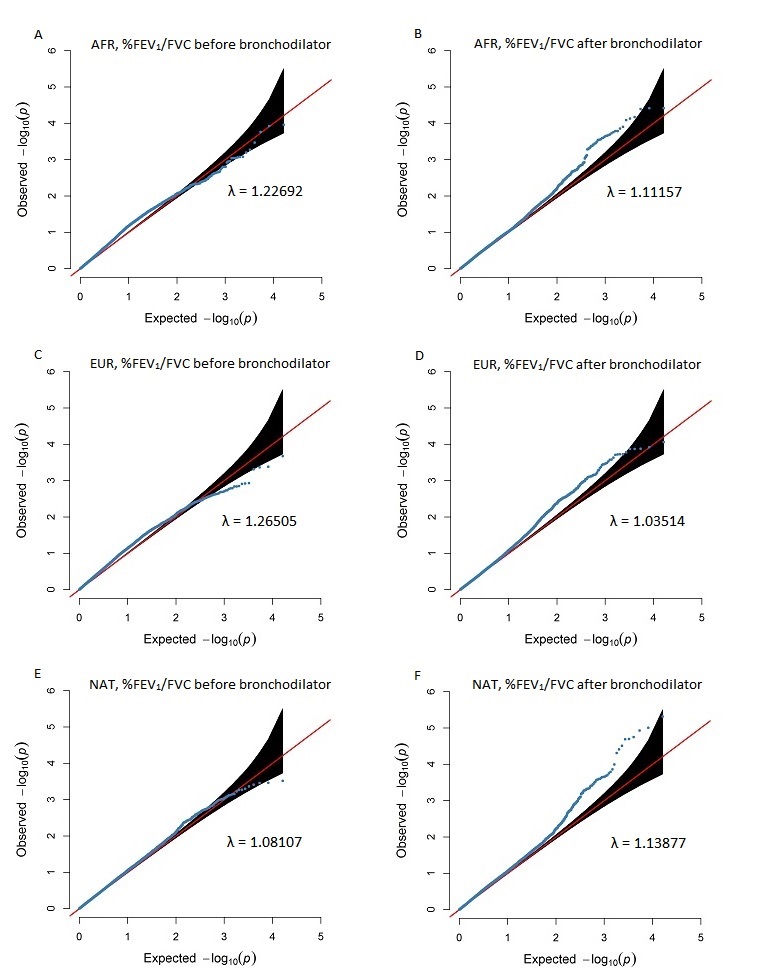

Supplement: Supplementary file 1 [file genes-11-01047-s001.zip › Figure S2.jpeg]

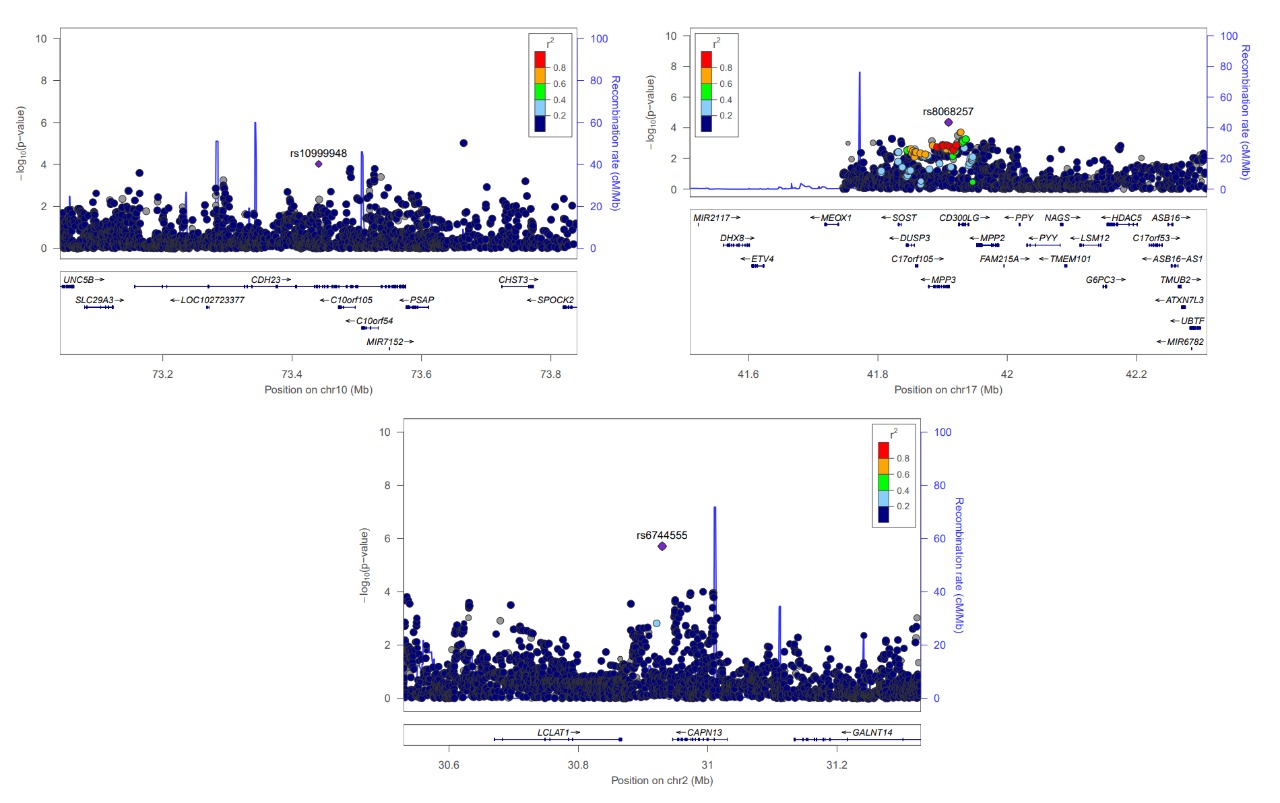

Supplement: Supplementary file 1 [file genes-11-01047-s001.zip › Figure S3.jpeg]

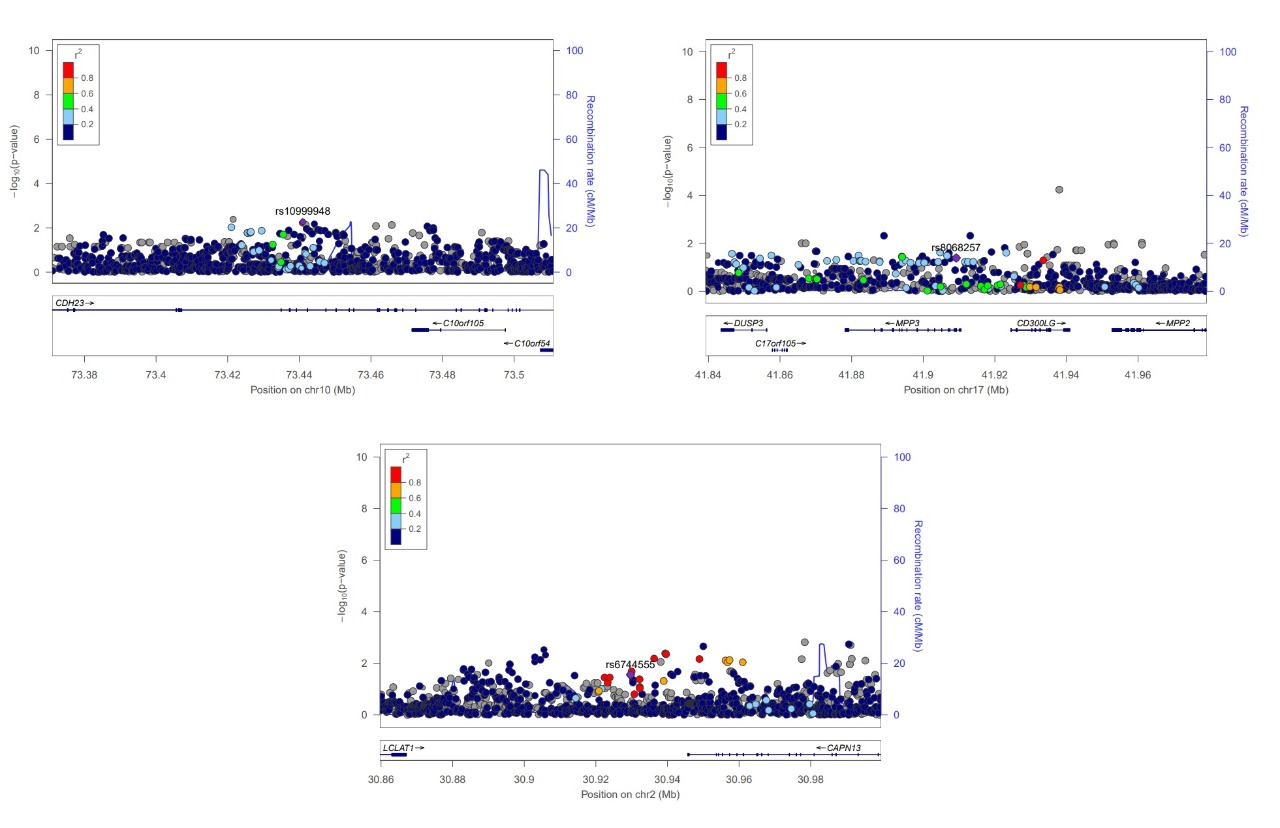

Supplement: Supplementary file 1 [file genes-11-01047-s001.zip › Figure S4.jpeg]

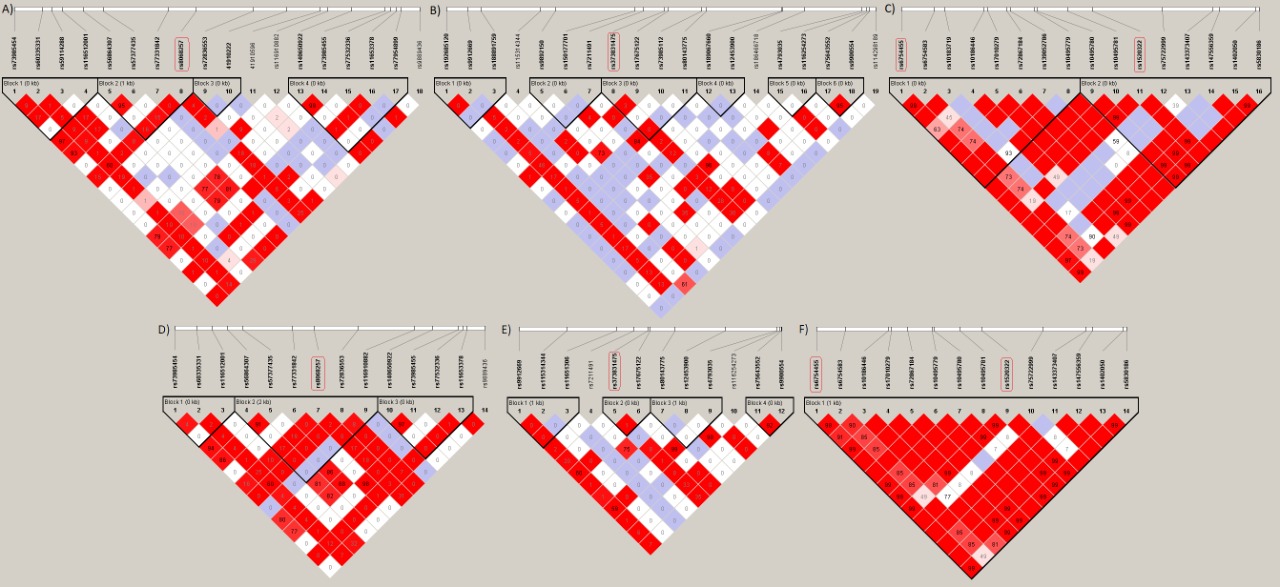

Supplement: Supplementary file 1 [file genes-11-01047-s001.zip › Figure S5.jpeg]

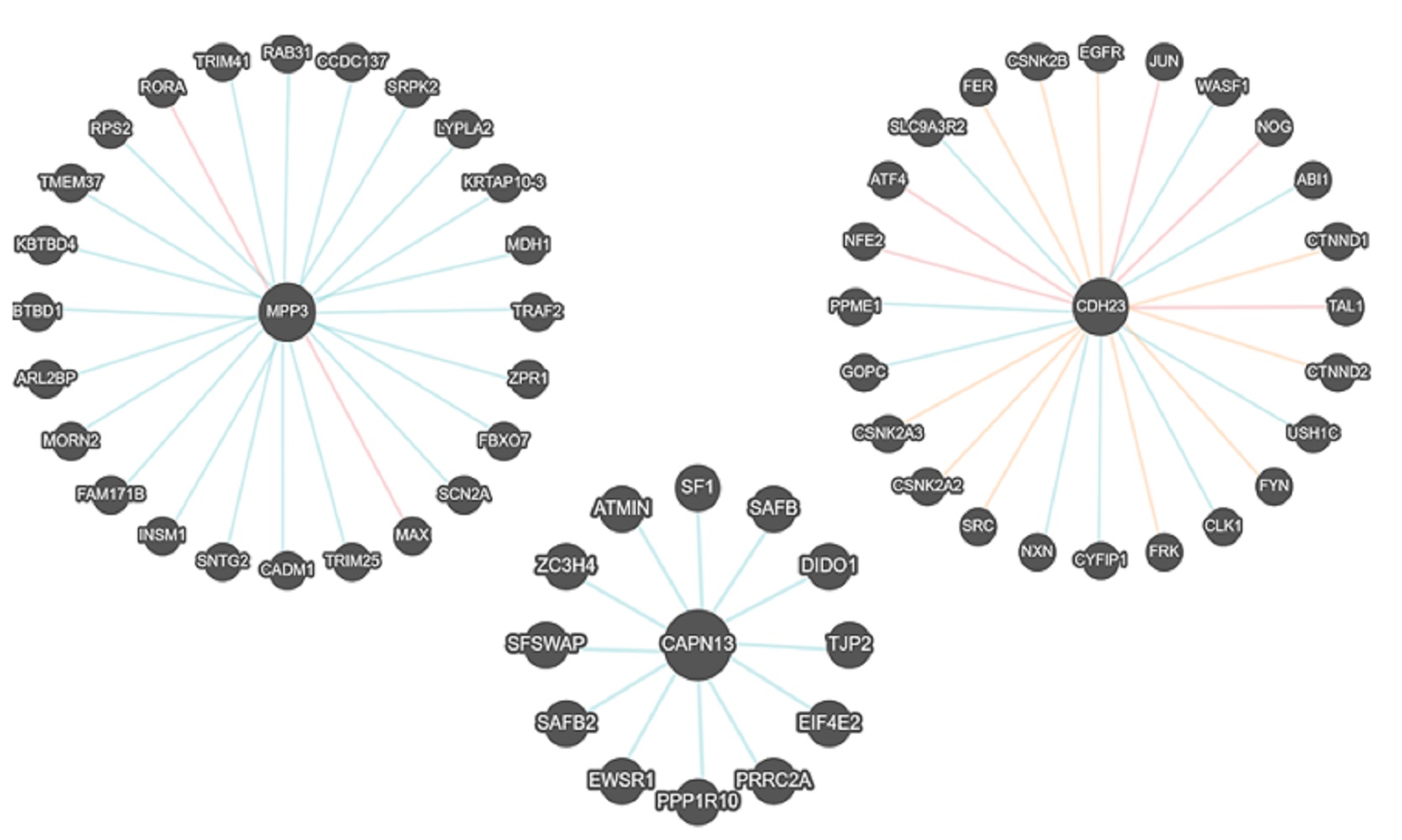

Supplement: Supplementary file 1 [file genes-11-01047-s001.zip › Figure S6.jpg]
